# Supplementary material for: Call for emergency action to limit global temperature increases, restore biodiversity, and protect health
Source: Lancet Reg Health Am. 2021 Sep 5;2:100070. doi: 10.1016/j.lana.2021.100070 (PMC8418896; doi:10.1016/j.lana.2021.100070)
Supplement: Supplementary file 1 [file mmc1.docx]

Appendix

**Call for emergency action to limit global temperature increases, restore biodiversity, and protect health**

The Comment Call for emergency action to limit global temperature increases, restore biodiversity, and protect health is being publish simultaneously in the following journals. This full list can also be found here, as well as a further list of supporting journals: [https://www.bmj.com/content/full-list-authors-and-signatories-climate-emergency-editorial-september-2021](https://nam11.safelinks.protection.outlook.com/?url=https%3A%2F%2Fwww.bmj.com%2Fcontent%2Ffull-list-authors-and-signatories-climate-emergency-editorial-september-2021&data=04%7C01%7Cstephanie.clague%40lancet.com%7C759a85bf19974e15a7aa08d9616e5fbb%7C9274ee3f94254109a27f9fb15c10675d%7C0%7C0%7C637647948954160809%7CUnknown%7CTWFpbGZsb3d8eyJWIjoiMC4wLjAwMDAiLCJQIjoiV2luMzIiLCJBTiI6Ik1haWwiLCJXVCI6Mn0%3D%7C1000&sdata=eL8bFyXlLHDFLPHXnk%2FSV%2BudC64%2FPwWDtd9LL%2FHt%2Fzk%3D&reserved=0)

*Acta Orthopaedica et Traumatologica Turcica*

*Advances in Nursing Science*

*Advances in Nutrition*

*African Journal of Laboratory Medicine*

*Afro-Egyptian Journal of Infectious and Endemic Diseases*

*Age and Ageing*

*Alcohol and Alcoholism*

*Allergy*

*Alpha Psychiatry*

*American Journal of Clinical Pathology*

*American Journal of Health-System Pharmacy*

*American Journal of Hypertension*

*American Society of Microbiology*

*Animal Bioscience*

*Annals of African Surgery*

*Annals of Behavioral Medicine*

*Annals of Oncology*

*Annals of Public Health*

*Annals of the Rheumatic Diseases*

*Annals of the Royal College of Surgeons of England*

*Archives of Disease in Childhood*

*Archives of the Turkish Society of Cardiology*

*Asia Pacific Journal of Public Health*

*Balkan Medical Journal*

*Belgian Journal of Medicine*

*Biosis: Biological Systems*

*BJOG*

*BMJ Case Reports*

*BMJ Evidence-Based Medicine*

*BMJ Global Health*

*BMJ Health & Care Informatics*

*BMJ Innovations*

*BMJ Leader*

*BMJ Military Health*

*BMJ Nutrition, Prevention & Health*

*BMJ Open*

*BMJ Open Gastroenterology*

*BMJ Open Ophthalmology*

*BMJ Open Quality*

*BMJ Open Respiratory Research*

*BMJ Open Science*

*BMJ Open Sport & Exercise Medicine*

*BMJ Paediatrics Open*

*BMJ Quality & Safety*

*BMJ Sexual & Reproductive Health*

*BMJ Supportive & Palliative Care*

*BMJ Surgery, Interventions, & Health Technologies*

*Bosnian Journal of Basic Medical Sciences*

*Brain*

*Brain Communications*

*British Dental Journal*

*British Journal of General Practice*

*British Journal of Ophthalmology*

*British Journal of Sports Medicine*

*British Medical Bulletin*

*Bulletin of the World Health Organization*

*Cadernos de Saúde Pública*

*Canadian Journal of Respiratory Therapy*

*Canadian Medical Association Journal*

*Cardiovascular Research*

*Caribbean Medical Journal*

*Chinese Science Bulletin*

*CIN: Computers, Informatics, Nursing*

*Clinical Medicine*

*Croatian Medical Journal*

*Crohn's & Colitis 360*

*Cureus Journal of Medical Science*

*Current Developments in Nutrition*

*Danish Medical Journal*

*Diseases of the Colon & Rectum*

*Dutch Journal of Medicine*

*East African Medical Journal*

*EBioMedicine*

*EClinicalMedicine*

*Emergency Medicine Journal*

*EP Europace*

*European Heart Journal*

*European Heart Journal - Acute Cardiovascular Care*

*European Heart Journal - Cardiovascular Imaging*

*European Heart Journal - Case Reports*

*European Heart Journal - Digital Health*

*European Heart Journal - Quality of Care and Clinical Outcomes*

*European Heart Journal – Cardiovascular Pharmacotherapy*

*European Journal of Cardio-Thoracic Surgery*

*European Journal of Cardiovascular Nursing*

*European Journal of Hospital Pharmacy*

*European Journal of Preventive Cardiology*

*European Journal of Public Health*

*Evidence-Based Mental Health*

*Evidence-Based Nursing*

*Family Medicine and Community Health*

*Family Practice*

*Finnish Medical Journal*

*Frontline Gastroenterology*

*Gaceta Sanitaria*

*Gastrointestinal Nursing*

*General Psychiatry*

*Global Health Action*

*Global Heart*

*Health Policy and Planning*

*Health Promotion International*

*Health Promotion Journal of Australia*

*Heart*

*Huisarts en wetenschap*

*Human Molecular Genetics*

*Human Reproduction*

*IJQHC Communications*

*Indian Journal of Medical Ethics*

*Indian Journal of Medical Research*

*Inflammatory Bowel Diseases*

*Injury Prevention*

*Innovation in Aging*

*Integrated Healthcare Journal*

*International Journal of Epidemiology*

*International Journal of Gynaecology & Obstetrics*

*International Journal of Gynecological Cancer*

*International Journal of Health Policy and Management*

*International Journal of Integrated Care*

*International Journal of Medical Students*

*International Journal of Nursing Studies*

*International Journal of Older People Nursing*

*International Journal of Pharmacy Practice*

*International Nursing Review*

*JAMIA Open*

*JMIR Public Health & Surveillance*

*JNCI Cancer Spectrum*

*Journal of Child Health Care*

*Journal of Clinical Pathology*

*Journal of Crohn’s and Colitis*

*Journal of Epidemiology & Community Health*

*Journal of Health and Caring Sciences*

*Journal of Health, Population and Nutrition*

*Journal of Medical Ethics*

*Journal of Medical Genetics*

*Journal of Medical Imaging and Radiation Sciences*

*Journal of Nepal Paediatric Society*

*Journal of Neurology Neurosurgery & Psychiatry*

*Journal of Open Health Data*

*Journal of Pharmaceutical Health Services Research*

*Journal of Pharmacy and Pharmacology*

*Journal of Public Health*

*Journal of Surgical Case Reports*

*Journal of Surgical Protocols and Research Methodologies*

*Journal of the American Medical Informatics Association*

*Journal of the Medical Association of Thailand*

*Journal of the National Cancer Institute*

*Journal of the Norwegian Medical Association*

*Journal of the Royal Society of Medicine*

*Journal of Travel Medicine*

*Journal of Tropical Pediatrics*

*Journal of Turkish Society of Microbiology*

*Kafkas Universitesi Veteriner Fakültesi Dergisi*

*Khyber Medical University Journal*

*La Revista Universitas Medica*

*Lab Medicine*

*Medical Humanities*

*Medical Journal of Australia*

*Medical Mycology*

*Medwave*

*Nephrology Dialysis Transplantation*

*Neuro-Oncology Advances*

*Neuro-Oncology Practice*

*Neurology*

*New England Journal of Medicine*

*Nicotine & Tobacco Research*

*Nurse Author & Editor*

*Nursing Inquiry*

*Nutrition Reviews*

*Occupational and Environmental Medicine*

*Occupational Medicine*

*Oxford Open Climate Change*

*Oxford Open Immunology*

*Pacific Rim Journal of International Nursing Research*

*Paediatrics & Child Health*

*Palliative Medicine*

*Pan American Journal of Public Health*

*Pediatric Infectious Disease Society of the Philippines Journal*

*Pediatric Nursing*

*Pharmaceutical Journal*

*PLOS Medicine*

*Postgraduate Medical Journal*

*Psychiatry and Clinical Psychopharmacology*

*PTJ: Physical Therapy & Rehabilitation Journal*

*Revista de la Facultad de Medicina Humana*

*Revista de Saúde Pública*

*Rheumatology*

*RMD Open*

*Schizophrenia Bulletin*

*Schizophrenia Bulletin Open*

*Sexually Transmitted Infections*

*SLEEP*

*SLEEP Advances*

*Stroke and Vascular Neurology*

*The American Journal of Clinical Nutrition*

*The BMJ*

*The Gerontologist*

*The Journal of Climate Change and Health*

*The Journal of Nutrition*

*The Journals of Gerontology, Series A*

*The Lancet*

*The Lancet Child & Adolescent Health*

*The Lancet Global Health*

*The Lancet Microbe*

*The Lancet Planetary Health*

*The Lancet Psychiatry*

*The Lancet Public Health*

*The Lancet Regional Health - Americas*

*The Lancet Regional Health - Europe*

*The Lancet Regional Health - Western Pacific*

*The National Medical Journal of India*

*The Pan-American Journal of Ophthalmology*

*Thorax*

*Tobacco Control*

*Translational Behavioral Medicine*

*Tropical Journal of Pharmaceutical Research*

*Turkish Archives of Otorhinolaryngology*

*Turkish Archives of Pediatrics*

*Turkish Journal of Anaesthesiology and Reanimation*

*Turkish Journal of Biochemistry*

*Turkish Journal of Cardiovascular Nursing*

*Turkish Journal of Orthodontics*

*Turkish Thoracic Journal*

*Veterinary Record*

*VOICE*

*Western Journal of Emergency Medicine*

*Women’s Healthcare: A Clinical Journal for NPs*

*World Journal of Pediatric Surgery*
